# The Development of a Mobile Health App for Breast Cancer Self-Management Support in Taiwan: Design Thinking Approach

**DOI:** 10.2196/15780

**Published:** 2020-04-30

**Authors:** I-Ching Hou, Min-Fang Lan, Shan-Hsiang Shen, Pei Yu Tsai, King Jen Chang, Hao-Chih Tai, Ay-Jen Tsai, Polun Chang, Tze-Fang Wang, Shuh-Jen Sheu, Patricia C Dykes

**Affiliations:** 1 School of Nursing National Yang-Ming University Taipei Taiwan; 2 Institute of Biomedical Informatics National Yang-Ming University Taipei Taiwan; 3 Department of Nursing National Taiwan University Hospital Taipei Taiwan; 4 Department of Computer Science and Information Engineering National Taiwan University of Science and Technology Taipei Taiwan; 5 Taiwan Breast Cancer Foundation Taipei Taiwan; 6 Division of Plastic Surgery Department of Surgery National Taiwan University Hospital Taipei Taiwan; 7 Department of Surgery College of Medicine National Taiwan University Taipei Taiwan; 8 Center for Patient Safety Research and Practice Brigham and Women’s Hospital Boston, MA United States; 9 Department of Medicine Harvard Medical School Boston, MA United States

**Keywords:** breast cancer, mobile health application, self-management, design thinking

## Abstract

**Background:**

Evidence has shown that breast cancer self-management support from mobile health (mHealth) apps can improve the quality of life of survivors. Although many breast cancer self-management support apps exist, few papers have documented the procedure for the development of a user-friendly app from the patient’s perspective.

**Objective:**

This study aimed to investigate the information needs of Taiwanese women with breast cancer to inform the development of a self-management support mHealth app.

**Methods:**

A 5-step design thinking approach, comprising empathy, define, ideate, prototype, and test steps, was used in the focus groups and individual interviews conducted to collect information on the requirements and expectations of Taiwanese women with breast cancer with respect to the app. A thematic analysis was used to identify information needs.

**Results:**

A total of 8 major themes including treatment, physical activity, diet, emotional support, health records, social resources, experience sharing, and expert consultation were identified. Minor themes included the desire to use the app under professional supervision and a trustworthy app manager to ensure the credibility of information.

**Conclusions:**

The strengths of the design thinking approach were user-centered design and cultural sensitivity. The results retrieved from each step contributed to the development of the app and reduction of the gap between end users and developers. An mHealth app that addresses these 8 main themes can facilitate disease self-management for Taiwanese women with breast cancer.

## Introduction

### Background

The term *breast cancer* refers to a malignant tumor that develops from cells in the breast. Its stage is usually expressed on a scale of 0 through IV with stage 0 describing noninvasive cancers that remain within their original location and stage IV describing invasive cancers that spread outside the breast to other parts of the body [1]. According to the World Health Organization, the estimated number of newly diagnosed breast cancer cases worldwide was 2,088,849 in 2018 [2], which was the highest among women’s cancers. Over 10,000 women are diagnosed with breast cancer every year in Taiwan. Most women are between the ages of 40 and 65 years at the time of diagnosis and usually have breast cancer that is between stages 0 and II [3]. Treatments for breast cancer include surgery, chemotherapy, immunotherapy, radiation therapy, hormonal therapy, and targeted therapy; these are often accompanied by side effects such as pain, fever, diarrhea, lymphedema, dry skin, fatigue, and insomnia [4]. Research indicates that there is compelling evidence of an increased risk of anxiety, depression, suicide, and neurocognitive and sexual dysfunctions among breast cancer survivors when compared with women with no prior incidence of cancer [5-7]. However, when breast cancer is treated appropriately, the 5-year survival rate approaches 88% [8]. Therefore, breast cancer is now treated as a chronic disease and self-management of symptoms is necessary for women to live well with breast cancer. Self-management is “the ability of the individual, in conjunction with family, community, and health care professionals, to manage symptoms, treatments, lifestyle changes, and psychosocial, cultural, and spiritual consequences of health conditions” [9]. Evidence suggests that self-management interventions improve the quality of life of women with breast cancer by enabling self-care [10], mindfulness-based stress reduction [11], and improved management of medical, emotional, and role-based tasks [12].

Today, mobile phones are popular and convenient, with an open architecture that allows third parties to develop mobile health (mHealth) apps. mHealth apps are highly relevant to the future of disease and health management [13]. Research indicates that using mHealth apps to support patients with breast cancer has many advantages including enhanced knowledge, increased physical activity, eliciting short-term reductions in weight, decreased anxiety, improved self-confidence, emotional well-being, and improved quality of life [14-17]. According to one study, there were 599 breast cancer–related apps in the iOS and Android markets in the United States in February 2016. Breast cancer–related apps most commonly addressed disease and treatment information (29.22%), addressed disease management (19.03%), and increased awareness (15.03%) [18].

### Objectives

To our knowledge, there are no breast cancer self-management support apps targeting Taiwanese women, and this creates several barriers in the use of existing apps. First, most apps do not provide educational content in traditional Chinese language. The noneffective use of language decreases app acceptance [19]. Second, when software is used to support instant translation, the accuracy and validity of the translation is unknown [20]. Third, the content of most existing apps lacks academic sources and references [18], leading to a lack of trust among Taiwanese women [21]. In addition to these barriers, several cross-cultural comparison studies have indicated that there are important differences in the depressive symptoms and quality of life between eastern and western breast cancer survivors [22-24]. Therefore, the contents of disease self-management support apps should consider cultural differences related to medical resources, food intake, personal beliefs, and support systems of their end users. To address this gap, our research team consisting of health care professionals (IC, MF, and PY), technology experts (SS and PC), and professionals at the Taiwan Breast Cancer Foundation (TBCF; KJ, HC, and IJ) partnered with Taiwanese women with breast cancer to investigate their information needs and to develop an app to meet their needs.

## Methods

### Study Design

In this study, our team used the design thinking approach. Design thinking is a user-centered problem-solving methodology that starts with assessing people's needs and then seeks innovative solutions to address the range of issues identified. The 5-step design thinking model comprises empathy, define (the problem), ideate, prototype, and test steps and was proposed by the Hasso-Plattner Institute of Design at Stanford (d.school) [25,26]. The concepts underpinning each step and the methodologies we used are summarized in Table 1. The study was approved by the Institutional Review Board (IRB) at National Yang-Ming University (IRB no: YM106005E).

We conducted this research in 2 phases using the 5-step design thinking methodology (Table 1). In phase one, we designed three concurrent 3-hour focus groups in the multifunction room of the TBCF, which included steps 1 through 3. We then entered the second phase of prototyping (step 4) followed by developing and testing (step 5). The research group conducted a data analysis by triangulating the qualitative interview data with our literature review and team discussion to iteratively develop the prototype. The original participants were invited to use the developed prototype, and it was validated further through in-depth interviews.

**Table 1:** Description of concepts and methodologies of the 5-step design thinking process.

Steps	Description of concepts	Methodologies
1. Empathy	To understand the way women with breast cancer do things and why, their physical and emotional needs, and what is meaningful to them	Seek to understand Icebreaker game Empathy by asking “needs for cancer fighting journey”
2. Define	To bring clarity and focus to the design. The goal is to craft a meaningful and actionable problem statement	Brainstorm by asking “the needs for the cancer journey” Write needs in post-it notes Name and prioritize the needs
3. Ideate	To concentrate on idea generation and get innovative solutions for women with breast cancer	Sketch the mockups of the app on mobile phone cardboards Demonstrate how to use the app Choose the favorite mockups
4. Prototype	To generate the demonstrative solution that can talk to women with breast cancer without investing a lot of time and money	Prototype system analysis Design the simulation app with computer-aided software Install in Android mobile phone
5. Test	To get feedback on the prototype from women with breast cancer and then find the right level of optimization of the prototype and solution	Provide the simulation app for trial use Feedback by asking “Tell me your feel to the app” statement

### Sampling and Data Collection

We used purposive sampling to enroll Taiwanese women with breast cancer in our study. As suggested by others, we planned to include 5 to 8 members in each focus group [27-29]. Therefore, we planned to recruit 15 to 24 women with breast cancer for 3 concurrent focus groups. The recruitment was posted on TBCF’s social networking sites (eg, Facebook and Line) and interested participants contacted us through online registration. A total of 20 women with breast cancer were registered but 5 dropped out because of illness or work obligations that coincided with our study date. The demographic data of the remaining 15 participants are shown in Table 2. Their average age was 55 years, and most women received routine outpatient care for follow-up or long-term hormone therapy. In the beginning of the focus group discussion, study investigators introduced the study, secured informed consent, and encouraged participants to share their ideas with us. In total, 2 female authors (IC and MF) moderated and 3 nurse graduate students joined each subgroup as a facilitator. In addition, 1 nurse graduate student and 2 TBCF staff observed, took photos, and recorded notes. Audio and visual recorders were used to collect data during the focus group discussion.

All participants in phase one were invited to participate in individual interviews. A total of 13 focus group subjects agreed to be interviewed individually. Two subjects did not enroll because of a transportation problem (ID: A5) and one felt that her disease was not severe enough to share her experience (ID: B4). The in-person interviews were held for 1.5 to 2 hours by a study investigator (MF) at a place near the subjects’ house or office, or at a café. An audio recorder was used to collect the interview data. All subjects were given US $30 after participating in the research.

**Table 2:** Demographic data of subjects.

ID	Test step	Age (years)	Education	Marital status	Number of children	Occupation	Cancer stage	Treatment	Current treatment stage
A1	Yes	63	High school	Married	2	Housewife	III	M^a^+C^b^+R^c^+H^d^+T^e^	F/U^f^
A2	Yes	47	High school	Married	1	Electrical industry	I	M+H	F/U with H
A3	Yes	56	Junior college	Married	2	Babysitter retirement	I	M+C+H	F/U with H
A4	Yes	45	Graduate	Married	2	Culture and education industry	II	M+C+T	Under treatment
A5	No	52	High school	Widowed	2	Unemployed	II	M+C+R+H+T	Under treatment
A6	Yes	58	High school	Married	1	Textile industry	III	M+C+R	F/U
B1	Yes	48	College	Widowed	0	Financial industry retirement	III	M+C+R+H	F/U with H
B2	Yes	41	Junior college	Married	1	Culture and education industry	III	M+C+R+H	F/U with H
B3	Yes	44	College	Single	0	Trade	II	M+C+R+H+T	F/U with H
B4	No	59	Junior college	Married	2	Housewife	0	B^g^+R	F/U
B5	Yes	63	College	Married	2	Culture and education industry	II	B+C+R+H+T	F/U with H
C1	Yes	68	College	Married	2	Retired	II	M+C+H	F/U with H
C2	Yes	61	College	Widowed	3	Department store clerk	III	M+C+R+H	F/U
C3	Yes	62	Junior college	Married	2	Retired	II	M+C+R	F/U
C4	Yes	63	Junior college	Married	1	Housewife	III	M+C+R+H	F/U with H

^a^M: modified radical mastectomy.

^b^C: chemotherapy.

^c^R: radiotherapy.

^d^H: hormone therapy.

^e^T: target therapy.

^f^F/U: follow-up.

^g^B: breast-conserving surgery.

### Procedure: Concepts and Study Activities

The concepts and study activities that were completed over 2 phases using the 5-step design thinking methodology are described below.

#### Phase One: Focus Group Approach

##### Step 1: Empathy

###### Concept

Empathy involves the work to understand and gain insight into people’s thoughts and needs, within the context of the design challenge. The designers’ goal is to understand how people do things and why they do so, their physical and emotional needs, how they think about their world, and what is meaningful to them [25].

###### Study Activity

**Figure 1:**
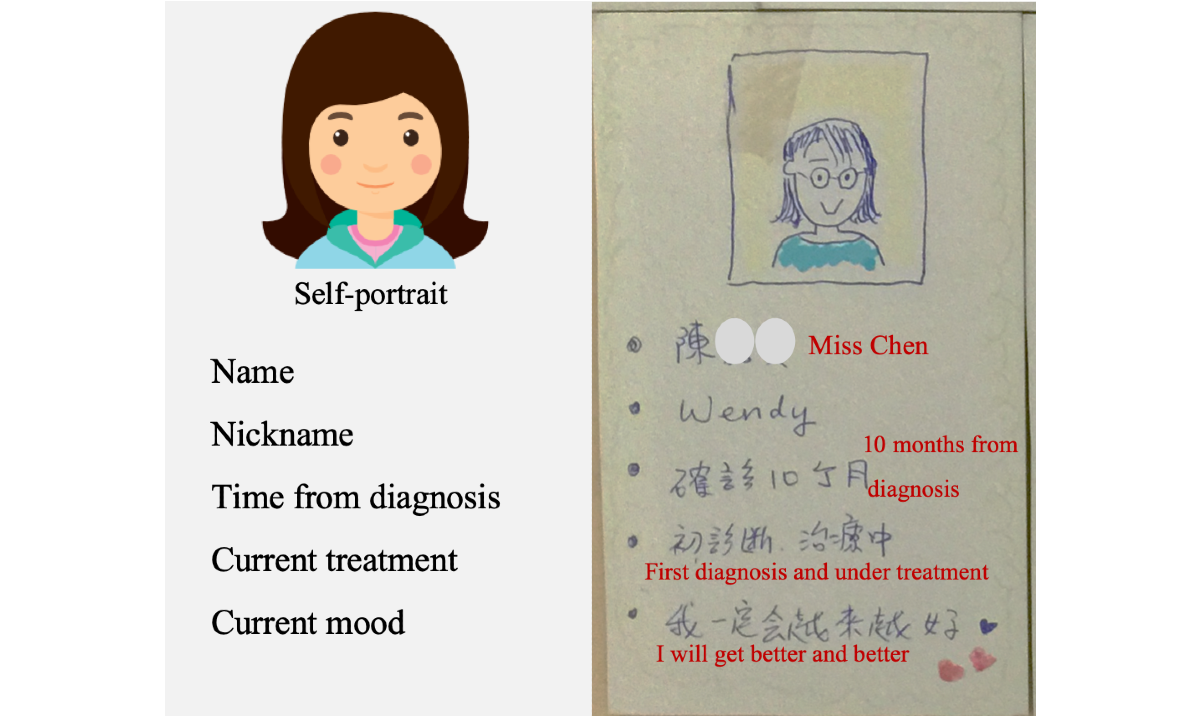
Self-instruction card for icebreaker. Demo card (left) and a participant’s card (right).

To understand breast cancer and the characteristics of Taiwanese women with breast cancer better, our team conducted a review of the literature and existing breast cancer apps. We also conducted interviews with the TBCF staff. In the beginning of the focus group discussion, each subject was asked to illustrate their own appearance and write their name, nickname, date of diagnosis, current treatment status, and their current mood on the card (Figure 1). The subjects then used the card to introduce themselves within each subgroup. This activity provided an opportunity for investigators to observe, watch, and listen to the subjects’ experiences at different stages of breast cancer treatment.

##### Step 2: Define

###### Concept

The define step in the design process brings clarity and focus to the design space. The goal is to craft a meaningful and actionable problem statement [25].

###### Study Activity

**Figure 2:**
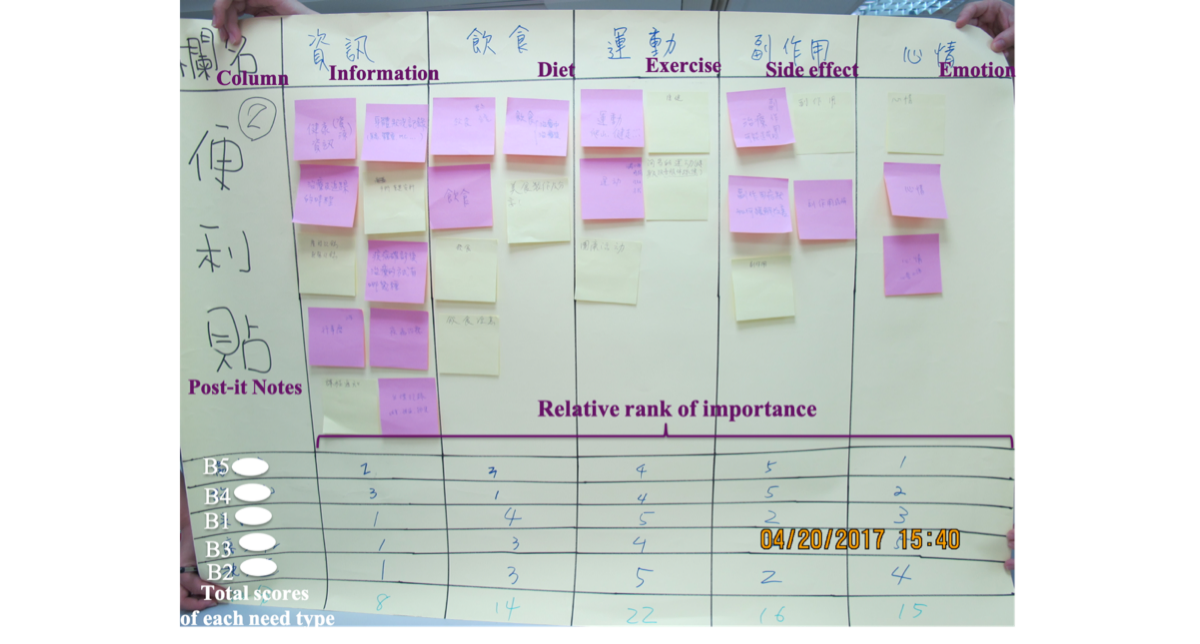
Names and prioritization of needs from subgroup B.

After a debriefing, subjects were asked to brainstorm on one question: “How may we use the mHealth app to support you through your cancer fighting journey?” Each need was written on a post-it note, and then notes with similar needs were arranged into the same column on a large poster. To ideate in response to information needs, each subgroup was asked to name the needs with notes similar to those created in the define step. Then, each participant of the subgroup was asked to rank the importance of their information needs using Arabic numbers (eg, 1 was the most important, 2 was the second most important, and so on). The facilitator of each group summarized the total score of each need type and its relative rank of importance (Figure 2).

##### Step 3: Ideate

###### Concept

The ideate step in the design process concentrates on idea generation. Ideation provides the fuel and is the source for building prototypes and putting innovative solutions in the hands of users [25].

###### Study Activity

**Figure 3:**
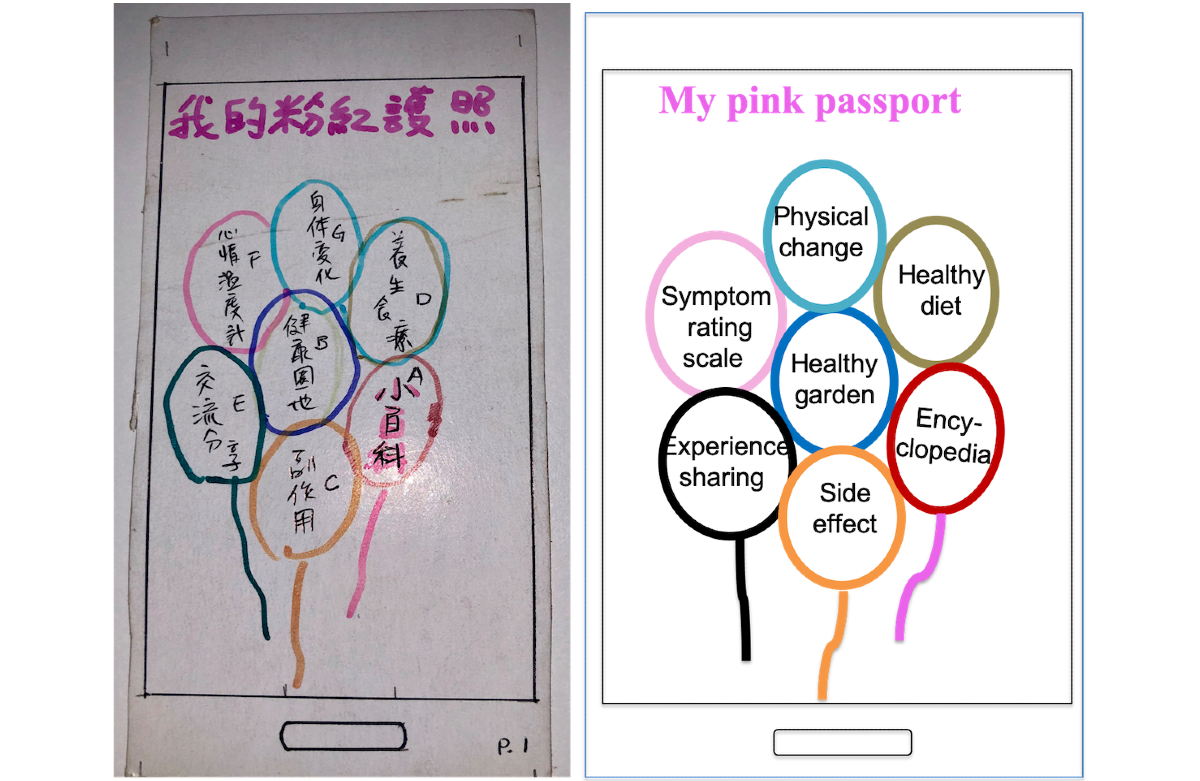
Mockups of the app from subgroup A (partial example). The original mockup menu of the app (left) and the same redrawn in English (right).

According to the prioritization determined in step 2, each subgroup was asked to sketch an mHealth app interface on 6.1-inch smartphone cardboard mockups using colored pens (Figure 3). At the end of the focus group discussion, the 3 subgroups were invited to demonstrate how they would use these mockups for disease self-management. After the demonstration, the participants were asked to choose their favorites. The mockups of subgroup A were preferred by most participants and were therefore used as a reference in the prototype step (step 4).

#### Phase Two: Individual Operation and Interview

##### Step 4: Prototype

###### Concept

The prototype step involves the iterative generation of artifacts intended to answer questions that get closer to a final solution**.** Through prototyping, designers can talk to users, resolve differences, reduce poor communication, and test ideas without investing a lot of time and money in programming [25].

###### Study Activity

**Figure 4:**
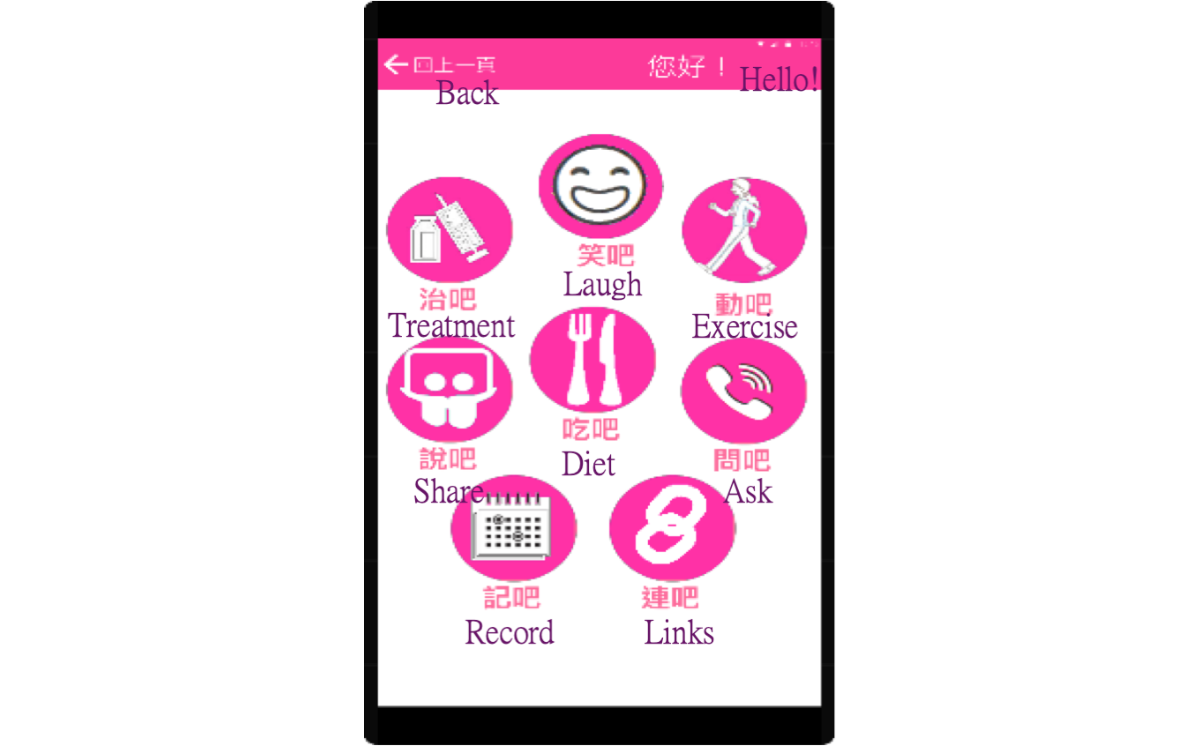
Prototype of the simulation app (partial example).

According to the results from steps 1 through 3, nursing informatics graduate student investigators (MF and PY) with basic mobile app programming skills, used the JustInMind Prototyper tool (JustInMind) to design the mHealth app simulation in the 6.1-inch, 1280×720-pixel touch screen (L×W×H: 161.5×84.5×9.3 mm) and an Android 4.2.2 Jelly Bean (Google LLC) mobile phone to be used as the tool in step 5 (Figure 4).

##### Step 5: Test

###### Concept

The test step aims to solicit feedback through the demonstration of the prototype to end users. This helps identify opportunities for the improvement and optimization of the prototype and solution [25].

###### Study Activity

To obtain participants’ feedback, individual interviews were held to evaluate the simulated breast cancer mHealth app. A study investigator (MF) showed the simulation to the participants and encouraged them to use it for about 15 min. The open-ended question, “Tell me your recommendations for each function in this simulated app,” was asked to collect feedback from each participant. An audio recorder was used during individual interviews, and the recordings were transcribed within 48 hours. A guideline for transcription was used to avoid inconsistency in transcript styles. The transcripts were emailed to each participant to confirm content accuracy.

###### Data Analysis

We adopted Norwell’s 6-phase methodology for a trustworthy thematic analysis to summarize our subjects’ information needs [30]. The first involved becoming familiar with the data. Subjects’ notes, naming of information needs, prototype cards, and individual interview transcripts were transcribed into an Excel spreadsheet. The text was read several times by 2 coders (MF and IC) to familiarize themselves with the data and to confirm accuracy. The second was generating initial codes. As the mockups from subgroup A were most preferred by participants, their naming of information needs was used as the initial codes to help guide the analysis. Texts that were similar to the initial codes were merged with those codes. Texts that could not be categorized into existing codes were formed into new ones based on their meanings. Two coders checked and finalized all the codes according to the semantic meaning to validate them, and to eliminate redundant ones. The third was searching for themes. The final codes were extracted for each theme to consider whether they formed a coherent pattern. The fourth involved reviewing themes. All themes were vetted during research team meetings. The fifth entailed defining and naming themes. The consensus among research team members was used to define and name each theme. The sixth involved producing the report. The Consolidated Criteria for Reporting Qualitative Research reporting guidelines [31] were used to produce this report. This process continued until the saturation of main themes was attained [32,33]. No new themes were found both after using this process and after 30 research team discussions were conducted over a year. Multimedia Appendix 1 shows an example of “diet” as a theme and the related codes that emerged from the thematic analysis in this study [34].

## Results

### Thematic Analysis

**Figure 5:**
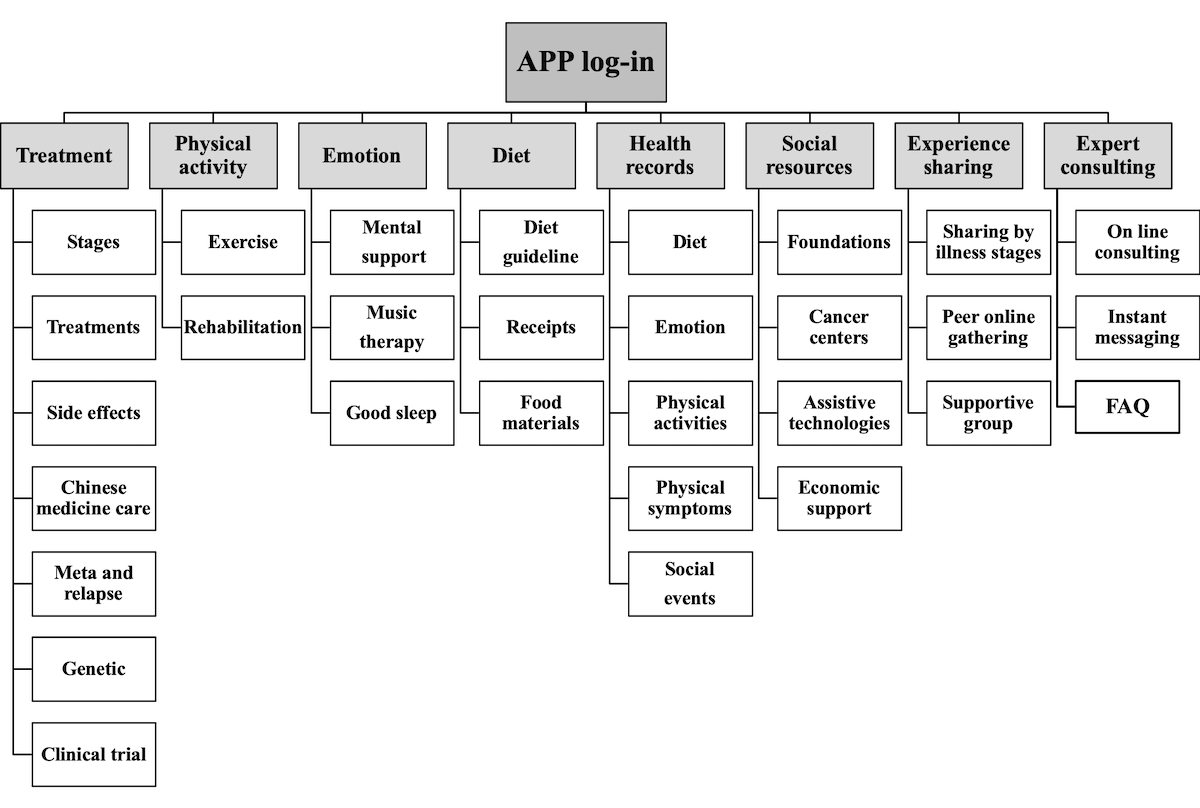
Framework of Breast Cancer Self-Management Support Mobile Health app.

After the thematic analysis, 8 major themes and 30 codes were identified. The framework is shown in Figure 5. The definitions of each theme and their codes are described below.

#### Theme 1: Treatment

Treatment was defined as information needs related to breast cancer treatment. A total of 31 notes (eg, “treatment protocol” and “the side effects of each treatment”) from the define step and 10 recommendations from the test step were categorized into the treatment theme. The codes included treatment stages, side effects, Chinese medicine care, metastases, and relapse, and genetic and clinical drug trials. Our subjects wanted more information on the treatment and side effects through the app:

We have one list, there are many (chemotherapy)drugs on it. (It tells) which one would make you lose hair and which one will not. I think it was very helpful and I had posted this list on our group four to five times.

B5, 63-year-old, stage II

The Chinese medication care code received the most feedback from the test step. Most subjects wanted to know which Chinese herbs were safe, when to receive Chinese medication support and care, and information on physicians qualified in Chinese medicine. A few subjects did not think that Chinese medicine care should be included in the app because they believed that it may interfere with formal treatment:

About the Chinese medication, I felt it was helpful, they can help you to relieve the uncomfortable symptoms. But you need to determine which Chinese medicine physician was good for you.

C4, 63-year-old, stage III

I believe in the Chinese medication, but my oncology physician told me not to take them because he thought the herbs might interfere with the hormone therapy.

A6, 58-year-old, stage III

#### Theme 2: Physical Activity

Physical activity was defined as the appropriate body movement and intensity for post–breast cancer treatment. A total of 24 notes (eg, “exercise,” “dancing,” “rehabilitation,” and “Qi Gong”) from the define step and 20 recommendations from the test step were categorized into the physical activity theme. The codes included exercise and rehabilitation. Most subjects identified preferred exercises and societies (eg, TBCF, Formosa Cancer Foundation, and Taiwan Cancer New Life Association) or private fitness firms (eg, Curves) for these exercises. Some recommended contacting a Taiwanese physical therapist to get his demonstration video for breast cancer exercises for use through the app.

We noted that some subjects described schedule conflicts with desired exercise activities. Others said that they would not go out during chemotherapy for fear of infection:

I would like to participant (Infinite Youth), but the time was too early to participant.

A6, 58-year-old, stage III

I would not want to participant in the course because I am afraid of being infected from the close space.

B1, 48-year-old, stage III

#### Theme 3: Emotion

Emotion was defined as psychological support during breast cancer treatment. A total of 20 notes (eg, “emotion,” “mental support,” and “entertainment”) from the define step and 11 recommendations from the test step were categorized into the emotion theme. The codes included mental support, music therapy, and sleeping well. For mental support, there were some short encouraging statements added to the simulation app. Some subjects expressed that they wanted to keep these statements in their mobile phone for easy access and use them for spiritual sustenance:

Can I download it here? Then I will save as my (mobile phone) desktop.

A2, 47-year-old, stage I

All participants believed that music in the simulation app could relieve stress related to their disease. Some subjects were choristers of TBCF. They recommended using their songs in the future app to help other patients who could not routinely participate in the chorus because of their geographical location:

The songs of the TBCF chorus could be provided (in this App) and let other patients listen to them. In fact, we hope that the patients during the treatment can come out (eg, participate the chorus). Some of sisters lived too far away to participant (the chorus). You cannot ask the them to come only for 1-hour chorus activity because it takes 2 to 3 hours in traffic to get there (eg, TBCF multifunction room).

C2, 61-year-old, stage III

To sleep well, the subjects shared that mindfulness, acupuncture point massage, and the pressure release activity were helpful:

I download the mindfulness activities in my phone. They were very helpful for me to practice.

C3, 62-year-old, stage II

#### Theme 4: Diet

Diet was defined as nutrition support and the norm for healthy foods for post–breast cancer treatment. A total of 18 notes (eg, “diet,” “diet guideline,” “gourmet food,” and “food and nutrition”) from the define step and 14 recommendations from the test step were categorized into the diet theme. The codes included diet guidelines, recipes, and ingredients. Most subjects reported that they could not prepare food by themselves during cancer therapy because of physical weakness. They were also concerned about what they can or cannot eat during treatment. Therefore, they recommended the inclusion of recipes from specific publications and recommended a recipe-sharing function on the app. The subjects also requested a convenient and reliable food ordering and delivery function on the app:

My sister prepared food for me. I was very weak during treatment.

A2, 47-year-old, stage I

Can I eat the beef? Some sister told me not to eat (beef).

A6, 58-year-old, stage III

It is good to share their own recipes.

B1, 48-year-old, stage III

It was convenient that the food can be delivered home when it is not convenient to go out during chemotherapy.

A1, 63-year-old, stage III

#### Theme 5: Health Records

Health records were defined as a diary of self-reported information related to breast cancer treatment and self-management. A total of 18 notes (eg, “body weight record,” “body temperature record,” and “arm circumference”) from the define step and 25 recommendations from the test step were categorized into the health records theme. The codes included the food diary, emotions, physical activity, physical symptoms, and social events. For the food diary, some subjects mentioned that they had participated in the nutrition training course. The nutritionist had asked them to record their daily diet. Therefore, it was helpful for them to have these functions on the app. However, they looked forward to feedback from the nutritionist after recording their diet and uploading photos:

It was ok to upload the photo (to the nutritionist) but you need to have nutritionist.

A1, 63-year-old, stage III

The subjects shared that they felt negative emotions during their treatment and that they wanted to record their moods on the app. However, some believed that writing about their mood without reflection was insufficient. Others expressed that by recording their emotions on the app, patients could reflect on their negative thoughts, recover from bad moods, and remain focused on positive thinking:

*Only record the emotion is not enough. You do not make the reflection. You shouldpractice self-conversation while entry the emotion records.* [C3, 62-year-old, stage II]

As for physical activity records (eg, heart rate, time, frequency, reminders), half the subjects felt that it would be difficult to remember to record their data without an auto-recording device (eg, an intelligent wearable device with a heart rate monitor). They also thought that it would be helpful for the app to remind them to engage in some physical activity if they had not done so for a while:

Generally, I can’t know so many (data) without using auto (monitor)device to record.

A2, 47-year-old, stage I

I hope the App could remind me if I do not exercise for a long time. Sometime people would be lazy when stay at home.

A4, 45-year-old, stage II

As for the physical symptoms record (eg, body weight with BMI, temperature, upper arm circumference, symptoms, defecation, sleeping time, and menstrual period), our subjects reported that they had already recorded their body weight every day and appreciated help in the calculation of their auto BMI. One of the subjects hoped that the app could help them control their body weight. Some reported checking their body temperature at home for fear of fever. None of the participants were used to measuring their upper arm circumference. Participants also reported that they had other symptoms and had asked for app functionality that allowed them to record other symptoms that they were having (eg, diarrhea and paronychia). Recording sleep duration was not perceived as helpful in measuring the quality of sleep. Some subjects who were experiencing menopause felt that the menstrual period record was not necessary for them. However, younger patients felt that it was helpful to show their physician this record with the notes, as it helped predict the next date of menstruation:

I am afraid that my BW was increased after checking it. Hopefully, it can have the function to tell us how to control BW such as eating fewer starchy foods.

A1, 63-year-old, stage III

Sleep record was not necessary. Some will have more anxiety because they are not sleeping well.

C4, 63-year-old, stage III

To predict and record the next menstruation so we don’t need to use another application.

B3, 44-year-old, stage II

As for social event records (eg, date of treatment, clinic, examination, and social events), some subjects requested that we expand the social record to include a wider range of social events (eg, dancing and gardening society membership).

#### Theme 6: Social Resources

Social resources was defined as social or economic support during breast cancer treatment. A total of 13 notes (eg, “resource,” “underwear tailor,” and “cancer foundation”) from the define step and 11 recommendations from the test step were categorized into the social resources theme. The codes included foundations, cancer centers, assistive technologies, and economic support. Most subjects had experience joining breast cancer–related foundations in Taiwan. Some of them reported serving as volunteers and getting more information from these foundations. Only a few reported having experience contacting the cancer center at a hospital. When it came to assistive technologies, they also shared information such as how to get a free wig, hat, and compression sleeve. Three subjects also mentioned information on economic support. They expressed their desire to have information on how to access these types of resources:

I volunteer at three foundations. Being a volunteer made me better understand their resources.

A3, 56-year-old, stage I

Are there cancer centers in the north, middle, south, east Taiwan?

B1, 46-year-old, stage III

You can add the information (eg, to the app) for where to borrow a wig. There are many wigs at the Hope Foundation, and they have many different stylish wigs.

C4, 63-year-old, stage III

When one of the parents had cancer, their family could apply for the financial support. However, no patient knows this information.

A3, 56-year-old, stage I

#### Theme 7: Experience Sharing

Experience sharing was defined as peer support among women with breast cancer. A total of 10 notes (eg, “experience sharing,” “peer online gathering,” and “peer support”) from the define step and 3 recommendations from the test step were categorized into the experience sharing theme. The codes included experience sharing by illness stage, peer online gathering, and support groups. Every subject supported the function of experience sharing videos or online gathering on the app. For the experience sharing video, they preferred an audio recording rather than an actual one. For the online gathering, they used existing social network apps (eg, Line and Facebook). They identified management issues that should be addressed, such as preventing negative or false information. For the support group, all subjects agreed that they wanted to offer encouragement to other patients:

You can record the voice of your emotion when diagnosed with breast cancer without showing your face.

A1, 63-year-old, stage III

Everyone is using Line, Facebook, it will not be ok to use on line gathering only in this App except you can link to Line, Facebook. In addition, the on line gathering without management should avoid for someone had negative emotion or false information.

A4, 45-year-old, stage II

#### Theme 8: Expert Consulting

Expert consulting was defined as expert support during breast cancer treatment. A total of 5 notes (eg, “consulting,” “online clinic for wound,” and “professional team online response”) from the define step and 7 recommendations from the test step were categorized into the social resources theme.

The codes included professional online consulting, instant messaging, and frequently asked questions. For professional online consulting, they believed that questions could not be answered in real time because of the workload of the professionals. Some subjects felt that it would decrease their workload if the questions were categorized as frequently asked questions:

I am wondering the professionals behind the App will answer the same question frequently? If frequently asked questions could be read somewhere, it will decrease the percentage of repeated questions and also decrease the workload of the professionals.

A4, 45-year-old, stage II

## Discussion

### Principal Findings and the Differences From Previous Studies

According to our 5-step design thinking approach and thematic analysis, a total of 8 themes that included treatment, physical activity, emotions, diet, health records, social resources, experience sharing, and expert consulting were retrieved. In each theme, there were multiple codes that consisted of the information needs of the end users of the app. Previous research has indicated that the information needs of women with breast cancer included knowledge of the disease, the impact of breast cancer on the body, cancer metastasis or relapse, understanding and preparing for treatment, preventing and facing side effects and risk, survival prediction, survival rate, consultations, follow-up schedule, diet suggestion and restrictions, body image changes, self-management, and mental support [35-38]. Four of our main themes and the codes (eg, treatment, diet, emotional support, and health records) matched findings from prior research. In this study, our subjects did not mention the information needs of survival prediction and survival rate. It may have been that our subjects were mostly current breast cancer victims and were focused on healthy lifestyles to prevent cancer recurrence [39].

Previous research has also indicated that physical activity including exercise and physical therapy was helpful in lymphedema prevention, postoperative pain relief, and body weight control to prevent cancer relapse [39,40]. Our subjects expressed a positive attitude toward participation in group physical activities. However, in some cases, low immune tolerance and meeting schedules limited participation. Therefore, including some e-learning exercise courses on the app to enhance flexibility should be considered.

With regard to the theme of diet, our subjects’ perceptions were consistent with findings from previous studies that suggested that women with breast cancer were concerned about healthy eating, food, and nutrition-related side effects of chemotherapy [41-43]. The incorporation of culturally appropriate Taiwanese recipes for women with breast cancer and related nutritional information in the simulation app was positively received by most subjects. However, subjects also felt that it would be difficult to cook by themselves during treatment. Thus, offering some convenient and reliable food ordering and delivery functions on the app was also considered helpful. Diet and physical activity recommendations could help those who had completed treatment control their body weight. Participants requested more advanced and useful body weight control functions within the app such as food calorie or energy burning calculators.

We found that some subjects kept their own health records (eg, recoding body weight and temperature). The simulation app offered many structured health record options but based on the subjects’ feedback, it was difficult for us to assess whether Taiwanese women with breast cancer wanted to keep these records on their own in the future. According to a previous study based on a survey of oncology patients on app-assisted cancer care, the introduction of mobile apps needs to follow different strategies depending on the patients’ attitudes and apps could support clinic visits, document adverse effects, and provide reminders of treatment dates or medication schedules [44].

In summary, 8 main themes were identified by the research team. The information needs based on these main themes and their codes were used to form the framework for a breast cancer self-management support mHealth app. Most themes were consistent with previous studies but some new themes specific to Chinese culture were also uncovered. We believe that the design thinking approach is a strong method for identifying the information needs for Taiwanese women with breast cancer for the development of mHealth technology.

### Minor Findings

From the analysis, two minor findings emerged. The first was that the app should be used under the supervision of a professional. Taking physical activity for example, we provided three levels of rehabilitation actions to help women with breast cancer recover from surgery and to prevent lymphedema. However, they did not understand when to do these actions. Such patients would like to consult a professional rather than use an app. They also wanted the nutritionist to review their daily food records and prescribe the correct diet based on their assessment. We concluded that professional support while using the app would make end users more confident in the benefits of using the mHealth app.

The second minor finding was related to the management of the app. Expert consulting was seen as important for supporting women with breast cancer; however, such services will require a significant time commitment from professionals. Our subjects were concerned about the workload of these professionals and recommended that we offer frequently asked questions instead. Moreover, women with breast cancer wanted to share their experiences on online forums and also recommended using an app manager to ensure the credibility of information. These two minor findings can facilitate end user trust in the app.

### Design Thinking Approach in Developing a Comprehensive and Culturally Sensitive App

The 5-step design thinking approach allowed users to brainstorm, design mockups, test, and provide feedback before the actual breast cancer–related self-management support app was developed. Through this approach, the app developer can identify new app requirements and compare end user requirements with those addressed in existing apps. In the ideate step, our participants sketched several balloons with different function labels as the main menu of the app (Figure 3) on the smartphone prototype. In eastern culture, people use a circle to represent the notion that everything is copasetic. Women with breast cancer may use the notion to pray for a smooth process during their cancer treatment. The design thinking approach and use of the illustration activity helped discover the user’s interface preferences and increased the programmer’s sensitivity to their needs.

In this study, the women with breast cancer had some information needs related to traditional Chinese medicine (TCM). The role of TCM was viewed as supportive medicine in the treatment of breast cancer. TCM is commonly used in eastern countries (eg, in Chinese culture, people often use Chinese herbs such as *Angelica sinensis*, *Fructus lycii*, pilosulae, tuceahoe, and shitake to enhance immunity) [45,46]. We were unable to find references or existing breast cancer–related self-management support apps that provide evidence of the effects of TCM treatment. Consistent with previous Chinese studies [45,46], most of our subjects wanted to know how TCM could help them during treatment. However, most women with breast cancer in Taiwan are undergoing treatment in hospitals (eg, the western medicine approach) and most of them are told by their physicians that TCM may interfere with western medicine treatment (eg, cause drug-drug interactions). To prevent confusion, a few subjects rejected the idea of including TCM information in the app. For such information needs, our team plans to consult with professional TCM physicians in the future. Our study did show that the design thinking approach is adequate to develop a user-centered and culturally sensitive support app for patients with cancer in a different social context.

### Limitations

One limitation of this study was that the sample size was inadequate in offering perspectives from women across all stages of breast cancer (eg, initial diagnosis through stage IV). Our study was conducted in an urban area with more medical resources than are typically available in the more rural areas of the country, and this may result in a geographical bias. Our testing was done using a simulation app rather than the real app, which may result in different feedback.

### Summary and Conclusions

Our team used the design thinking process to retrieve information needs related to the use of an mHealth app by women with breast cancer in Taiwan. The needs were retrieved using one focus group with three subgroups that included brainstorming, discussion, and validation. The interactive simulation app coupled with individual interviews helped us identify content and begin prototyping before designing the real app. A total of 8 themes with multiple codes consisting of common and culturally specific information needs provided the framework for the self-management support mHealth app that we developed for Taiwanese women with breast cancer.

## Appendix

Multimedia Appendix 1 Analysis of information needs framework (example).

## Abbreviations

IRB: Institutional Review Board
mHealth: mobile health
NTUST-NYMU: National Taiwan University of Science and Technology and National Yang-Ming University Joint Research Program
TBCF: Taiwan Breast Cancer Foundation
TCM: traditional Chinese medicine

## References

[1] Breast Cancer Information and Support.

[2] World Health Organization (2018). Cancer Today.

[3] Health Promotion Administration Ministry of Health and Welfare.

[4] Breast Cancer Information and Support.

[5] Carreira H, Williams R, Müller M, Harewood R, Stanway S, Bhaskaran K (2018). Associations between breast cancer survivorship and adverse mental health outcomes: a systematic review. J Natl Cancer Inst.

[6] Jacob L, Kalder M, Kostev K (2017). Incidence of depression and anxiety among women newly diagnosed with breast or genital organ cancer in Germany. Psychooncology.

[7] Patoo M, Moradi AR, Payandeh M (2014). P0134 Relationship between depression, anxiety, and quality of life in women with breast cancer. Eur J Cancer.

[8] Taiwan Cancer Registry.

[9] Richard AA, Shea K (2011). Delineation of self-care and associated concepts. J Nurs Scholarsh.

[10] van Dijck S, Nelissen P, Verbelen H, Tjalma W, Gebruers N (2016). The effects of physical self-management on quality of life in breast cancer patients: A systematic review. Breast.

[11] Eyles C, Leydon GM, Hoffman CJ, Copson ER, Prescott P, Chorozoglou M, Lewith G (2015). Mindfulness for the self-management of fatigue, anxiety, and depression in women with metastatic breast cancer: a mixed methods feasibility study. Integr Cancer Ther.

[12] Loh SY, Packer T, Chinna K, Quek KF (2013). Effectiveness of a patient self-management programme for breast cancer as a chronic illness: a non-randomised controlled clinical trial. J Cancer Surviv.

[13] Yu LP (2014). The development of the mobile medical industry and the opportunities in Taiwan - from the US mobile medical application guidelines. Reg Med News.

[14] Zhu J, Ebert L, Guo D, Yang S, Han Q, Chan SW (2018). Mobile breast cancer e-support program for Chinese women with breast cancer undergoing chemotherapy (part 1): qualitative study of women's perceptions. JMIR Mhealth Uhealth.

[15] Zhu J, Ebert L, Liu X, Wei D, Chan SW (2018). Mobile breast cancer e-support program for Chinese women with breast cancer undergoing chemotherapy (part 2): multicenter randomized controlled trial. JMIR Mhealth Uhealth.

[16] Pope Z, Lee JE, Zeng N, Lee HY, Gao Z (2019). Feasibility of smartphone application and social media intervention on breast cancer survivors' health outcomes. Transl Behav Med.

[17] McCarroll ML, Armbruster S, Pohle-Krauza RJ, Lyzen AM, Min S, Nash DW, Roulette GD, Andrews SJ, von Gruenigen VE (2015). Feasibility of a lifestyle intervention for overweight/obese endometrial and breast cancer survivors using an interactive mobile application. Gynecol Oncol.

[18] Giunti G, Giunta D, Guisado-Fernandez E, Bender J, Fernandez-Luque L (2018). A biopsy of Breast Cancer mobile applications: state of the practice review. Int J Med Inform.

[19] mHIMSS App Usability Work Group (2012). Amazon Web Services.

[20] Moshi MR, Tooher R, Merlin T (2018). Suitability of current evaluation frameworks for use in the health technology assessment of mobile medical applications: a systematic review. Int J Technol Assess Health Care.

[21] Team HON (2020). Health On the Net.

[22] Milbury K, Kavanagh A, Meng Z, Chen Z, Chandwani KD, Garcia K, Perkins GH, McQuade J, Raghuram NV, Nagarathna R, Liao Z, Nagendra HR, Chen J, Guo X, Liu L, Arun B, Cohen L (2017). Depressive symptoms and positive affect in Chinese and United States breast cancer survivors: a cross-cultural comparison. Support Care Cancer.

[23] Wen K, Fang CY, Ma GX (2014). Breast cancer experience and survivorship among Asian Americans: a systematic review. J Cancer Surviv.

[24] Fischer M, Inoue K, Matsuda A, Kroep JR, Nagai S, Tozuka K, Momiyama M, Weijl NI, Langemeijer-Bosman D, Ramai SR, Nortier JW, Putter H, Yamaoka K, Kubota K, Kobayashi K, Kaptein AA (2017). Cross-cultural comparison of breast cancer patients' Quality of Life in the Netherlands and Japan. Breast Cancer Res Treat.

[25] (2010). Stanford d.school.

[26] Mummah SA, Robinson TN, King AC, Gardner CD, Sutton S (2016). IDEAS (Integrate, Design, Assess, and Share): a framework and toolkit of strategies for the development of more effective digital interventions to change health behavior. J Med Internet Res.

[27] Morgan DL, McCracken G (1996). Focus Groups As Qualitative Research.

[28] Krueger RA (2002). Semantic Scholar.

[29] Lee T, Sheu S, Chang H, Hung Y, Tseng L, Chou S, Liang T, Liu H, Lu H, Chen M, Liu Y, Tsai C, Sun J (2019). Developing a web-based comic for newly diagnosed women with breast cancer: an action research approach. J Med Internet Res.

[30] Nowell LS, Norris JM, White DE, Moules NJ (2017). Thematic analysis: striving to meet the trustworthiness criteria. Int J Qual Methods.

[31] Tong A, Sainsbury P, Craig J (2007). Consolidated criteria for reporting qualitative research (COREQ): a 32-item checklist for interviews and focus groups. Int J Qual Health Care.

[32] Ando H, Cousins R, Young C (2014). Achieving saturation in thematic analysis: development and refinement of a codebook. Compr Psychol.

[33] Glaser BG, Strauss AL (1967). Discovery of Grounded Theory: Strategies for Qualitative Research.

[34] Forman HJ, Davies KJ, Ursini F (2014). How do nutritional antioxidants really work: nucleophilic tone and para-hormesis versus free radical scavenging in vivo. Free Radic Biol Med.

[35] Alba-Ruiz R, Bermúdez-Tamayo C, Pernett JJ, Garcia-Gutierrez JF, Cózar-Olmo JM, Valero-Aguilera B (2013). Adapting the content of cancer web sites to the information needs of patients: reliability and readability. Telemed J E Health.

[36] Lei CP, Har YC, Abdullah KL (2011). Informational needs of breast cancer patients on chemotherapy: differences between patients' and nurses' perceptions. Asian Pac J Cancer Prev.

[37] Liou Y, Zhao TT, Yuan CJ (2016). Qualitative study on information needs of different treatment stages for breast cancer patients. Nursing Journal Of Chinese Peoples Liberation Army.

[38] Jeang SR, Chi MT, Wang PH, Ku YC (2015). Process of Seeking Treatment for Newly Diagnosed Breast Cancer. VGH Nursing.

[39] Hayes SC, Johansson K, Stout NL, Prosnitz R, Armer JM, Gabram S, Schmitz KH (2012). Upper-body morbidity after breast cancer: incidence and evidence for evaluation, prevention, and management within a prospective surveillance model of care. Cancer.

[40] Merchant SJ, Chen SL (2015). Prevention and management of lymphedema after breast cancer treatment. Breast J.

[41] Kwok A, Palermo C, Boltong A (2015). Dietary experiences and support needs of women who gain weight following chemotherapy for breast cancer. Support Care Cancer.

[42] de Cicco P, Catani MV, Gasperi V, Sibilano M, Quaglietta M, Savini I (2019). Nutrition and breast cancer: a literature review on prevention, treatment and recurrence. Nutrients.

[43] Hou R, Wei J, Hu Y, Zhang X, Sun X, Chandrasekar EK, Voruganti VS (2019). Healthy dietary patterns and risk and survival of breast cancer: a meta-analysis of cohort studies. Cancer Causes Control.

[44] Kessel KA, Vogel MM, Kessel C, Bier H, Biedermann T, Friess H, Herschbach P, von Eisenhart-Rothe R, Meyer B, Kiechle M, Keller U, Peschel C, Schmid RM, Combs SE (2017). Mobile health in oncology: a patient survey about app-assisted cancer care. JMIR Mhealth Uhealth.

[45] Ching S, Mok E (2019). Adoption of healthy lifestyles among Chinese cancer survivors during the first five years after completion of treatment. Ethn Health.

[46] Cheng H, Sit JW, Cheng KK (2017). A qualitative insight into self-management experience among Chinese breast cancer survivors. Psychooncology.

