# Supplementary material for: The Development of a Mobile Health App for Breast Cancer Self-Management Support in Taiwan: Design Thinking Approach
Source: JMIR Mhealth Uhealth. 2020 Apr 30;8(4):e15780. doi: 10.2196/15780 (PMC7226037; doi:10.2196/15780)
Supplement: Multimedia Appendix 1 [file mhealth_v8i4e15780_app1.docx]

#### **Multimedia Appendix 1.** Analysis of information needs framework (example).

| Data from design thinking process | Codes | Theme |
| --- | --- | --- |
| *Brief notes*  Group A: Healthy food   - Food and nutrition - How to cook without losing nutrients? - How to eat without free radical reaction (eg, dietary with nutritional antioxidants) [34] - A video of recipes - How to choose food and how to clean food to remove pesticides   Group B: Diet   - The dietary considerations during chemotherapy - Recipes for chemotherapy meals - Gourmet food preparation   Group C: Diet   - Nutrition - Food ingredients - Cooking - The dietary considerations in any treatment stage   *Individual feedback*   - B1 (48-year-old, stage III): Homemade food should be added to the recipes. - B3 (44-year-old, stage II): The app can offer information on food ingredient websites including chemotherapy meals, farmers’ markets, etc. | *Principles of diet*   - Under treatment - Symptom release - Post treatment - Prevent relapse - Contraindications - Cooking methods   *Recipes*   - Cancer nutrition   *Food ingredients*   - Food ingredients website - Farmers’ markets - Special meals delivery service company for women undergoing chemotherapy | Diet |
